# Digital immunohistochemistry platform for the staining variation monitoring based on integration of image and statistical analyses with laboratory information system

**DOI:** 10.1186/1746-1596-9-S1-S10

**Published:** 2014-12-19

**Authors:** Aida Laurinaviciene, Benoit Plancoulaine, Indra Baltrusaityte, Raimundas Meskauskas, Justinas Besusparis, Daiva Lesciute-Krilaviciene, Darius Raudeliunas, Yasir Iqbal, Paulette Herlin, Arvydas Laurinavicius

**Affiliations:** 1 Department of Pathology, Forensic Medicine and Pharmacology, Faculty of Medicine, Vilnius University, Vilnius, Lithuania; 2 National Center of Pathology, affiliate of Vilnius University Hospital Santariskiu Clinics, Vilnius, Lithuania; 3 Path-Image/BioTiCla, University of Normandy, Unicaen, Caen, France

## Abstract

**Background:**

Digital immunohistochemistry (IHC) is one of the most promising applications brought by new generation image analysis (IA). While conventional IHC staining quality is monitored by semi-quantitative visual evaluation of tissue controls, IA may require more sensitive measurement. We designed an automated system to digitally monitor IHC multi-tissue controls, based on SQL-level integration of laboratory information system with image and statistical analysis tools.

**Methods:**

Consecutive sections of TMA containing 10 cores of breast cancer tissue were used as tissue controls in routine Ki67 IHC testing. Ventana slide label barcode ID was sent to the LIS to register the serial section sequence. The slides were stained and scanned (Aperio ScanScope XT), IA was performed by the Aperio/Leica Colocalization and Genie Classifier/Nuclear algorithms. SQL-based integration ensured automated statistical analysis of the IA data by the SAS Enterprise Guide project. Factor analysis and plot visualizations were performed to explore slide-to-slide variation of the Ki67 IHC staining results in the control tissue.

**Results:**

Slide-to-slide intra-core IHC staining analysis revealed rather significant variation of the variables reflecting the sample size, while Brown and Blue Intensity were relatively stable. To further investigate this variation, the IA results from the 10 cores were aggregated to minimize tissue-related variance. Factor analysis revealed association between the variables reflecting the sample size detected by IA and Blue Intensity. Since the main feature to be extracted from the tissue controls was staining intensity, we further explored the variation of the intensity variables in the individual cores. MeanBrownBlue Intensity ((Brown+Blue)/2) and DiffBrownBlue Intensity (Brown-Blue) were introduced to better contrast the absolute intensity and the colour balance variation in each core; relevant factor scores were extracted. Finally, tissue-related factors of IHC staining variance were explored in the individual tissue cores.

**Conclusions:**

Our solution enabled to monitor staining of IHC multi-tissue controls by the means of IA, followed by automated statistical analysis, integrated into the laboratory workflow. We found that, even in consecutive serial tissue sections, tissue-related factors affected the IHC IA results; meanwhile, less intense blue counterstain was associated with less amount of tissue, detected by the IA tools.

## Background

Digital immunohistochemistry (IHC) is one of the most promising applications brought by digital pathology, enabling new generation image analysis (IA) tools [1-3]. Robust and efficient digital IHC systems are expected to enable high throughput, accurate, and reproducible measurement of tissue markers, along with their spatial distribution. Conventional IHC routine is mostly based on qualitative and semi-quantitative visual evaluation of the tissue tested as well as tissue controls, to monitor the IHC staining quality. Multi-tissue controls on the same IHC slide can further improve the staining quality control [4]. While visual quality monitor is deemed sufficient for the conventional IHC, IA-based approach may require more sensitive monitoring in the digital IHC [5]. Although quantitative IA has been referred as valuable way to quantify staining intensity and assure day-to-day consistency of control tissue reactivity [6], we are not aware of published work on this aspect. We have previously shown that HER2 IHC multi-tissue controls, monitored by IA, reveal the staining intensity drifts and unexpected deviations undetected by routine slide-by-slide review by a pathologist [7]. Furthermore, data reduction by factor analysis has been helpful in retrieving hidden variation sources in IHC IA data [8] and could be useful in exploring quality indicators for digital IHC.

On the other hand, successful implementation of digital IHC depends on seamless integration of the IA and statistical analysis tools into pathology diagnosis and research workflow. Implementation, validation, calibration, continuous quality monitoring - all require swift quantitative feedback from the IA results. Digital IHC tissue control is a particular case, representing this efficiency need and possible solution scenarios.

We herewith present an automated system to monitor digital IHC multi-tissue controls, based on SQL-level integration of laboratory information system (LIS) with image and statistical analysis tools. The platform enables to explore hidden IHC staining variation factors in the serial sections of multi-tissue controls used in diagnostic IHC routine, based on multivariate analyses and visual representation of the IA results.

## Methods

**Figure 1:**
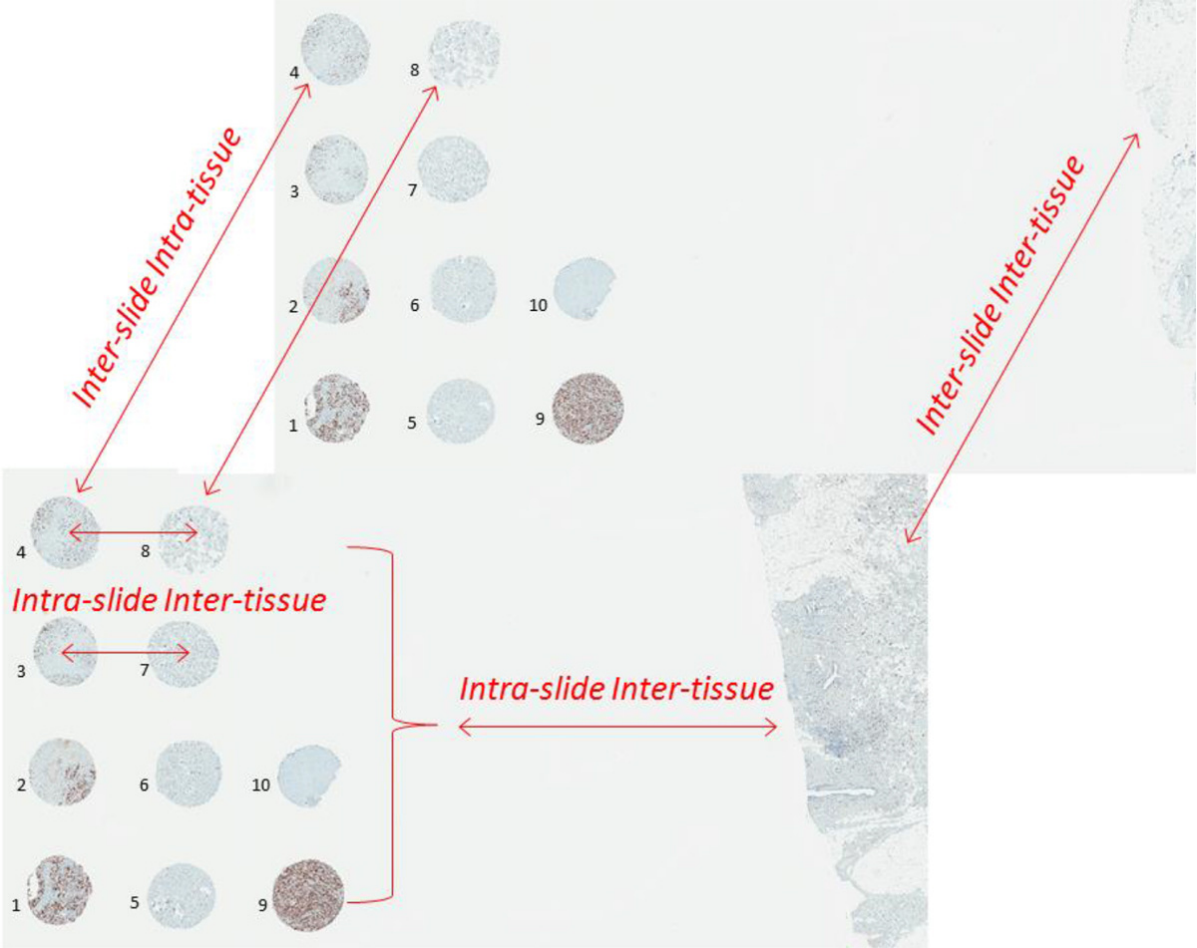
**The design of the TMA used as the IHC multi-tissue controls**. The cores are labeled with their sample identifiers used in this study. IA-detected variance between different tissue cores on the same slide is expected to reflect "intra-slide inter-tissue"variation, while IA-detected variance between consecutive sections of the same tissue core - "inter-slide intra-tissue" variance expected to reflect the variation of IHC staining properties overtime. The latter, if established, would then serve as "digital IHC control" to measure the test tissue "intra-slide inter-tissue" variance on the same slide.

IHC multi-tissue controls were constructed from paraffin blocks of breast cancer tissue with a broad range of Ki67 IHC positivity. Tissue microarrays (TMA) containing 10 tissue cores of 1 millimeter diameter were produced and consecutive serial sections were cut and stored at +4°C. Upon demand, when Ki67 IHC on breast cancer tissue were ordered by pathologist in a diagnostic routine, the slides with the multi-tissue control sections were used to add a section of a diagnostic sample. A unique and ascending barcode ID number was sent to the LIS by the Ventana Ultra machine when the slide label was printed at the microtome workstation, thus allowing retrieval of the serial section sequence from the LIS for further data management and integration with the IA results. After the IHC slides were routinely stained (Ultraview DAB detection kit on Ventana Ultra staining system (Ventana Medical Systems, Tucson, Arizona, USA; counterstained with Meyer's hematoxylin prepared in house), they were scanned (Aperio ScanScope XT, 20× objective magnification), TMA multi-tissue was assigned an appropriate Aperio TMALAB design, and IA algorithms were run on the control TMA spot images as well as on the test tissue whole slide images (WSI). SQL-based data integration ensured automated analysis of the TMA and WSI IA data by the SAS Enterprise Guide project, constructed to manage and analyze the IA data. Factor analysis and plot visualizations were performed to extract and monitor slide-to-slide variation of the Ki67 IHC staining characteristics, based on the sequence of serial multi-tissue sections identified by the Ventana label barcode ID in the LIS. Three TMA blocks were used in the study consequently until exhausted, to produce serial multi-tissue control sections (84, 31, and 69, respectively). Separate statistical analyses were carried out for each block; results from the third block are presented in the Results section. The control tissue samples (TMA cores) were labeled as represented in the Ki67 IHC spot images of the third TMA block (Figure 1); IA-detected variance between different tissue cores on the same slide is expected to reflect "intra-slide inter-tissue" variation, while IA-detected variance between consecutive sections of the same tissue core - "inter-slide intra-tissue" variance, expected to reflect the variation of IHC staining properties overtime. The latter, if established, would then serve as "digital IHC control" for the test tissue on the same slide.

The IA was performed by the Aperio/Leica Colocalization v.9 algorithm, tuned to extract brown and blue colors, as well as the Genie Classifier v.1/Nuclear v.9 algorithm, calibrated to enumerate Ki67-positive and negative tumour nuclear profiles in the breast cancer tissue [9]. Automated data management and statistical analysis workflow were achieved in the SAS Enterprise Guide v.5.1. Summary and variation statistics of the IA output variables were performed (Table 1). Factor analysis was carried out using factoring method of principal component analysis: factors were retained based on the threshold of the smallest eigenvalue of 1.0. Orthogonal varimax rotation of the initial factors was performed. A level of statistical significance was not set in this exploratory experiment. The LIS is a SQL and WEB-based system PathIS^®^, developed and maintained by the National Center of Pathology and the Baltic Information Technologies Institute, Vilnius, Lithuania.

**Table 1:** Slide-to-slide IHC staining variation of the 10 multi-tissue control samples, based on IA output variables.

SampleID	1	2	3	4	5	6	7	8	9	10
N	69	69	69	69	69	69	69	69	69	69
Mean										
Total Nuclei	2111	1006	859	1175	458	496	928	587	3059	120
Positive Nuclei	1436	541	310	400	74	89	122	111	2436	25
Percent Positive Nuclei	68	55	36	35	19	19	14	20	79	23
Positive Density	1462	895	752	616	285	270	250	373	1810	262
Negative Nuclei	674	464	550	775	384	406	806	476	623	95
Negative Density	694	749	1320	1160	1306	1178	1615	1504	464	882
Area of Analysis	0,97	0,60	0,41	0,66	0,26	0,32	0,50	0,31	1,34	0,09
Brown Intensity	110	138	133	126	174	174	171	163	106	183
Blue Intensity	167	171	170	170	173	172	170	170	171	179
Intensity Brown/Blue Ratio	0.66	0.81	0.78	0.74	1.01	1.01	1.01	0.96	0.62	1.03
Blue Area	0.10	0.12	0.13	0.18	0.18	0.18	0.20	0.14	0.12	0.09
Brown Area	0.36	0.22	0.07	0.09	0.05	0.05	0.05	0.04	0.61	0.02
Standard deviation										
Total Nuclei	527	454	275	380	423	393	487	377	524	162
Positive Nuclei	408	235	96	112	71	79	74	73	515	28
Percent Positive Nuclei	5	5	3	3	7	7	4	5	4	8
Positive Density	178	84	66	66	85	111	65	84	334	118
Negative Nuclei	164	232	183	273	357	326	422	310	106	136
Negative Density	103	148	136	102	321	244	214	233	74	290
Area of Analysis	0,22	0,24	0,13	0,21	0,22	0,22	0,27	0,19	0,07	0,10
Brown Intensity	3.61	4.83	5.85	5.32	2.51	3.03	2.27	4.61	5.80	2.98
Blue Intensity	3.21	2.86	3.50	2.75	3.27	3.99	4.08	4.35	2.53	3.51
Intensity Brown/Blue Ratio	0.02	0.03	0.04	0.04	0.02	0.02	0.03	0.04	0.03	0.02
Blue Area	0.03	0.02	0.03	0.04	0.04	0.04	0.03	0.03	0.02	0.08
Brown Area	0.12	0.09	0.02	0.02	0.02	0.02	0.02	0.01	0.10	0.02
Relative error										
Total Nuclei	0.25	0.45	0.32	0.32	0.92	0.79	0.52	0.64	0.17	1.35
Positive Nuclei	0.28	0.43	0.31	0.28	0.96	0.89	0.60	0.66	0.21	1.16
Percent Positive Nuclei	0.07	0.09	0.08	0.08	0.37	0.39	0.28	0.27	0.05	0.35
Positive Density	0.12	0.09	0.09	0.11	0.30	0.41	0.26	0.23	0.18	0.45
Negative Nuclei	0.24	0.50	0.33	0.35	0.93	0.80	0.52	0.65	0.17	1.42
Negative Density	0.15	0.20	0.10	0.09	0.25	0.21	0.13	0.15	0.16	0.33
Area of Analysis	0,22	0,39	0,31	0,31	0,84	0,69	0,53	0,61	0,06	1,14
Brown Intensity	0.03	0.04	0.04	0.04	0.01	0.02	0.01	0.03	0.05	0.02
Blue Intensity	0.02	0.02	0.02	0.02	0.02	0.02	0.02	0.03	0.01	0.02
Intensity Brown/Blue Ratio	0.04	0.04	0.05	0.05	0.02	0.02	0.03	0.05	0.05	0.02
Blue Area	0.27	0.15	0.27	0.22	0.25	0.22	0.13	0.22	0.17	0.88
Brown Area	0.33	0.39	0.31	0.25	0.41	0.38	0.32	0.35	0.16	0.65

## Results

**Figure 2:**
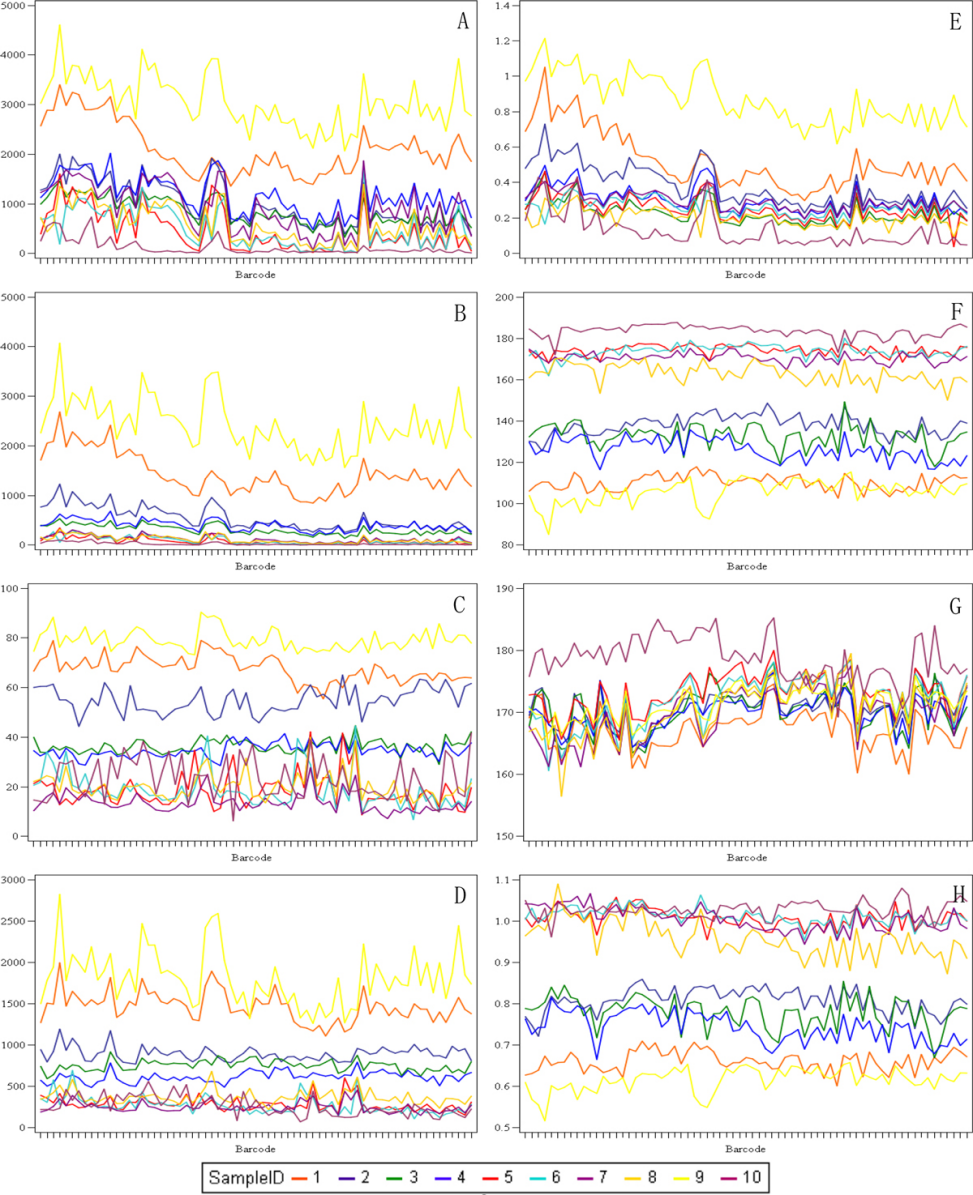
**Line plots representing slide-to-slide IHC staining variation of the TMA multicontrols**. The sequence of Ventana slide label ID is plotted as Barcode on the x axis to represent consecutive serial sections of TMA blocks of the 10 multi-tissue control cores (labelled as SampleID), based on image analysis results of: A. Total Nuclei; B. Positive Nuclei; C. Percent Positive Nuclei; D. Positive Density; E. Total Stained Area (mm^2^); F. Brown Intensity; G. Blue Intensity; H. Brown/Blue Intensity ratio.

Slide-to-slide IHC staining variation of the 10 multi-tissue control samples, represented by selected IA output variables, is presented in the Table 1 and Figure 2 (A, B, C, D and E, F, G, H plots represent data obtained by the Genie/Nuclear and Colocalization algorithms, respectively).

Firstly, rather significant intra-core variation can be noted in the variables reflecting the sample size of the spots (Total nuclei, Total stained area, Figure 2A, E): while continuous drift of these variables is likely to reflect tissue variability in the consecutive sections, the irregularities, often parallel in majority of the spots, may reflect tissue artefacts and/or staining variation. Indeed, inspection of the spot images with major abnormalities revealed presence of tissue artefacts.

Secondly, the variation of Ki67-positive nuclei detected in the consecutive sections was rather significant (Figure 2B, C); it was relatively more notable at the low end of scale (where main clinical interest is), also represented by higher relative error values in the cores with less Ki67 positivity (Table 1). To avoid potential impact of misdetection of negative tumour nuclei on the Ki67 positivity estimation, we calculated the "Positive Density" variable as the ratio of Ki67-positive nuclei to the Area of Analysis to be used in further analyses. Remarkably, the variation of the Positive Density appeared less aberrant at the low end of scale (Figure 2D), although this was not necessarily reflected by the Relative Error values compared to the Ki67-positive percent (Table 1).

Thirdly, the variation of the Brown and Blue Intensity as well as their ratio (Figure 2F, G, H) reflected inter-core variation dependent on the Ki67 positivity of the tumours sampled; however, the range of Blue Intensity inter-core variation was lower than that of the Brown Intensity. Intra-core variation of both Brown and Blue Intensity was rather low, while aberrant spot images revealed mostly tissue artefacts affecting the IA results.

Finally, since the multi-controls represent tissue samples from tumours with different Ki67 positivity, it is expected that the IA results on individual spots would reflect this; however, slide-to-slide variation of the same core would reveal continuous change due to some unavoidable tissue variation in the serial sections. Importantly, one can note the pattern that in some slides this variation appears parallel in most spots, while on other occasions it appears unrelated (Figure 1).

**Figure 3:**
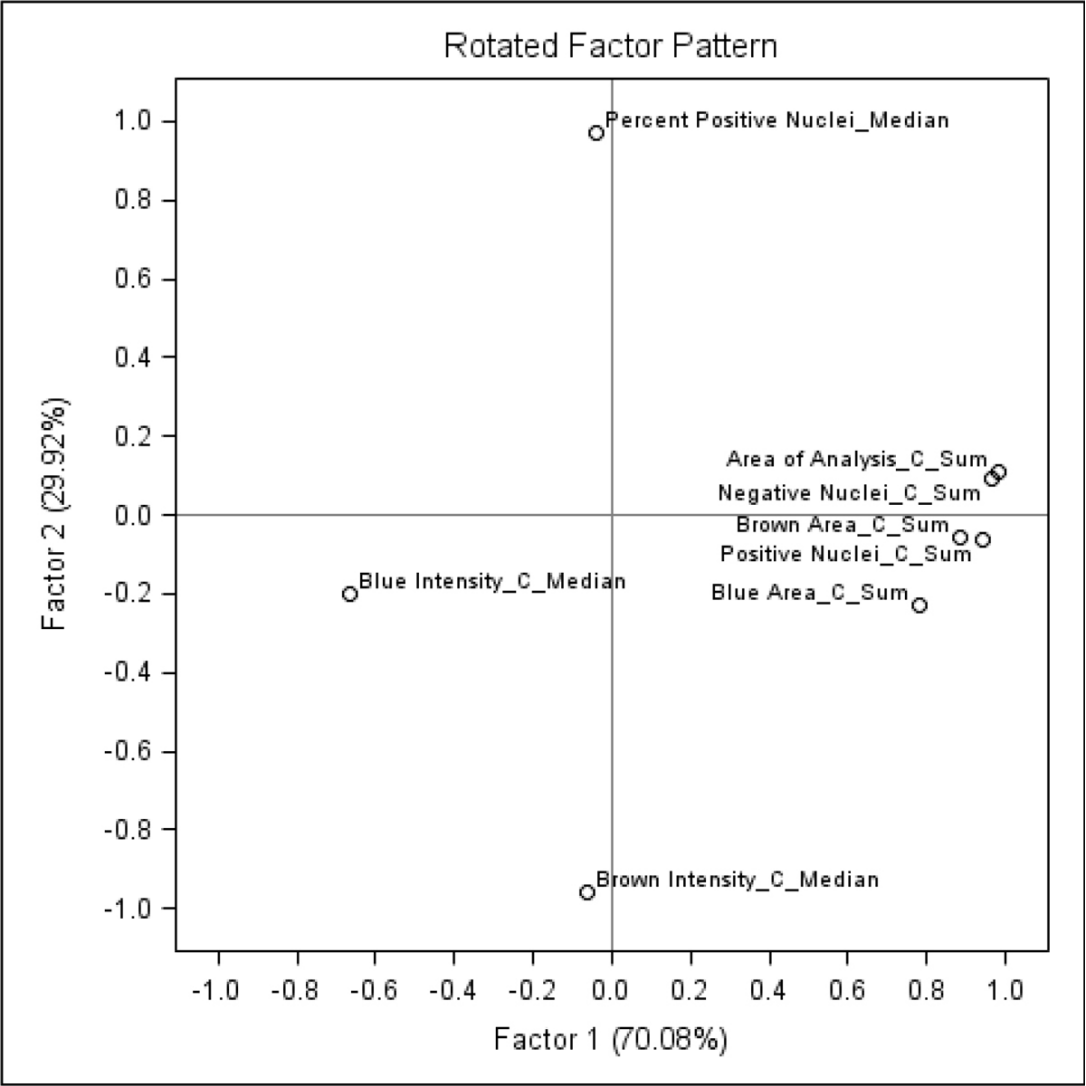
**Factor pattern representing parallel variance of the Colocalization and Genie/Nuclear algorithm output variables in aggregated image analysis data from the 10 TMA cores**. The variable loading plots: A. Factor-1 versus Factor-2; B. Factor-1 versus Factor-3.

**Figure 4:**
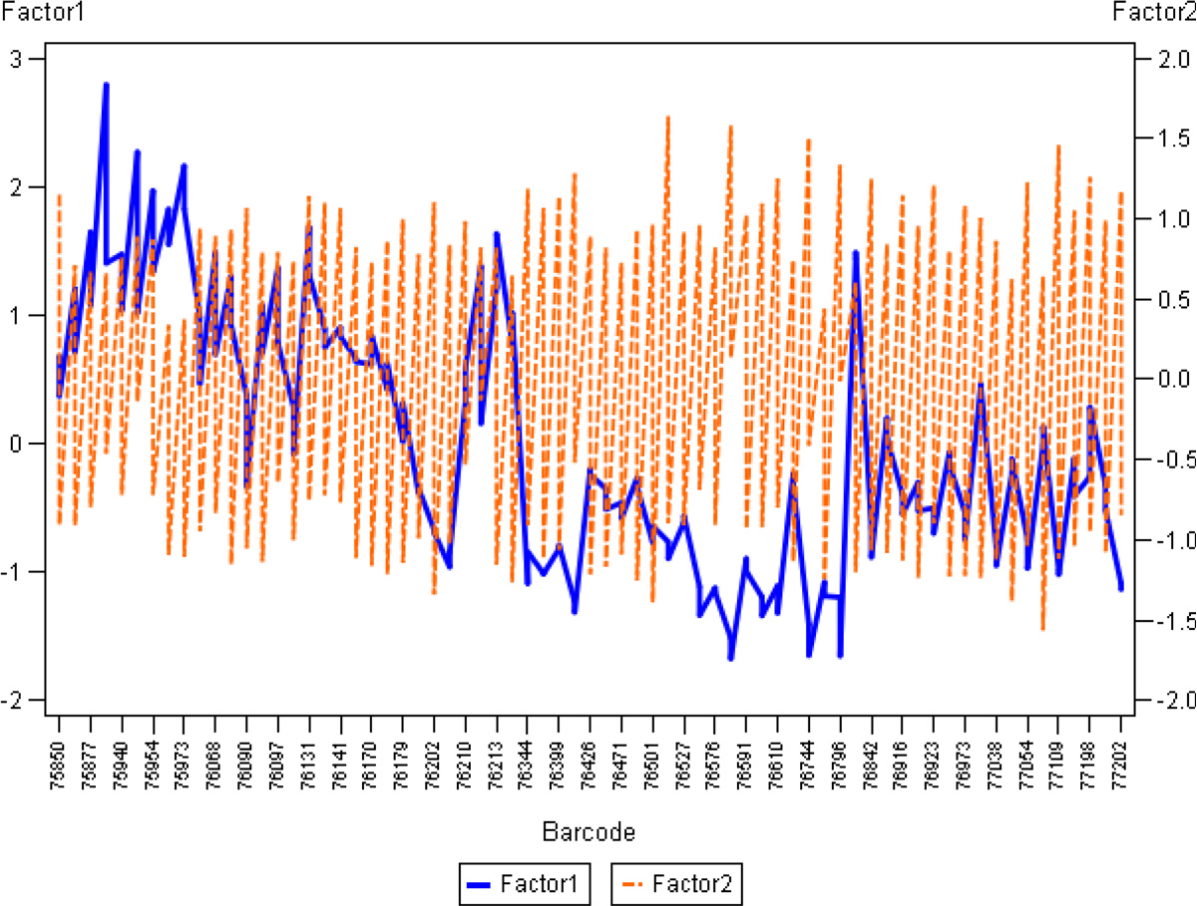
**Line plots representing slide-to-slide IHC staining variation of the Factor scores**. The factor scores generated from the analysis presented in Fig. 3 are plotted against the sequence of Ventana slide label ID (labelled as Barcode) on the x axis

**Figure 5:**
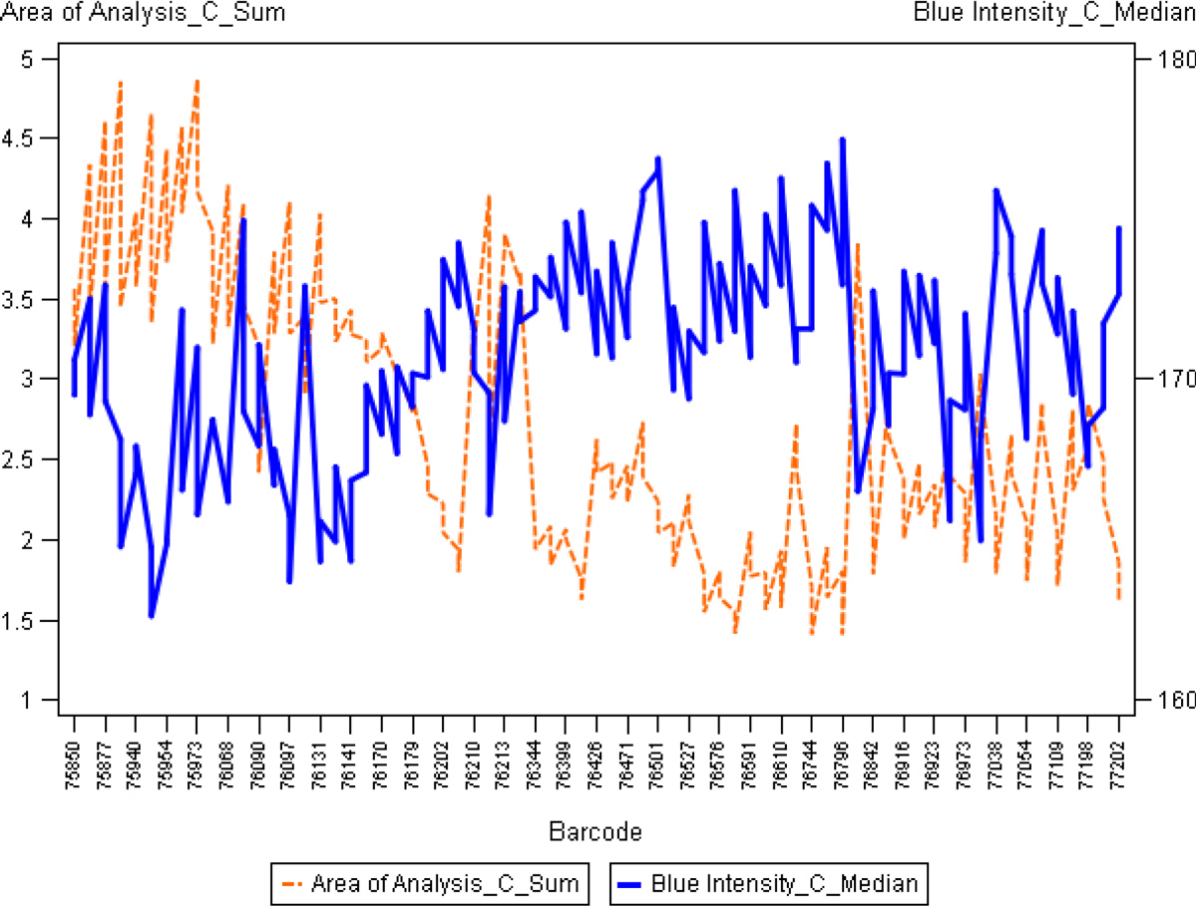
**Line plots representing slide-to-slide IHC staining variation of selected image analysis variables from aggregated TMA data**. Aggregated data (Median Blue Intensity and Area of Analysis) from image analysis of 10 TMA cores are plotted against the sequence of Ventana slide label ID (labelled as Barcode) on the x axis.

To further investigate potential sources of this variation, we have aggregated the IA results from the 10 cores as appropriate to represent them as one sample. Since the tissue-related variation in all of the 10 cores is expected to be random (except possible variation of the tissue section thickness and the slide scanning regime), aggregation of the data would represent a "super-sample" were tissue-related impact on the IA variance would be reduced. Therefore, variables like Median Blue Intensity, Total Stained area, Total Nuclei, would summarize parallel but disregard random variation of the individual core IA data. Factor analysis of the aggregated variables (Figure 3) revealed that the major source of variation (Factor 1) was characterized by positive loadings of the variables reflecting "sample size" detected by the IA algorithms: Blue Area and Brown Area by the Colocalization, and Area of Analysis, Positive Nuclei, Negative Nuclei by the Genie/Nuclear. Remarkably, the Factor 1 also revealed strong negative loading of Blue Intensity values (more intense blue correlated with more tissue detected by both algorithms). Meanwhile, the Factor 2 was represented by positive loadings of the Percent of Positive Nuclei and negative loadings of Brown Intensity (more intense brown correlated with higher Percent of Positive Nuclei). The factor pattern implies possible impact of tissue staining intensity variation on IA performance in terms of tissue detection, however, the percentage of positive nuclei is relatively independent of this effect (by definition, Factors 1 and 2 are linearly independent). To further demonstrate the relationships, the plots of the Factor 1 and 2 scores in the consecutive sections are presented in the Figure 4: while the Factor 2 scores reveal aberrant variation, the Factor 1 scores present notable drift with several peaks, potentially pointing to the IHC counterstain intensity changes, although impact of tissue-related factors cannot be ruled out. The peculiar relationship between the variables is also illustrated by the plot of Area of Analysis (detected by the Genie) and Blue Intensity (Figure 5).

**Figure 6:**
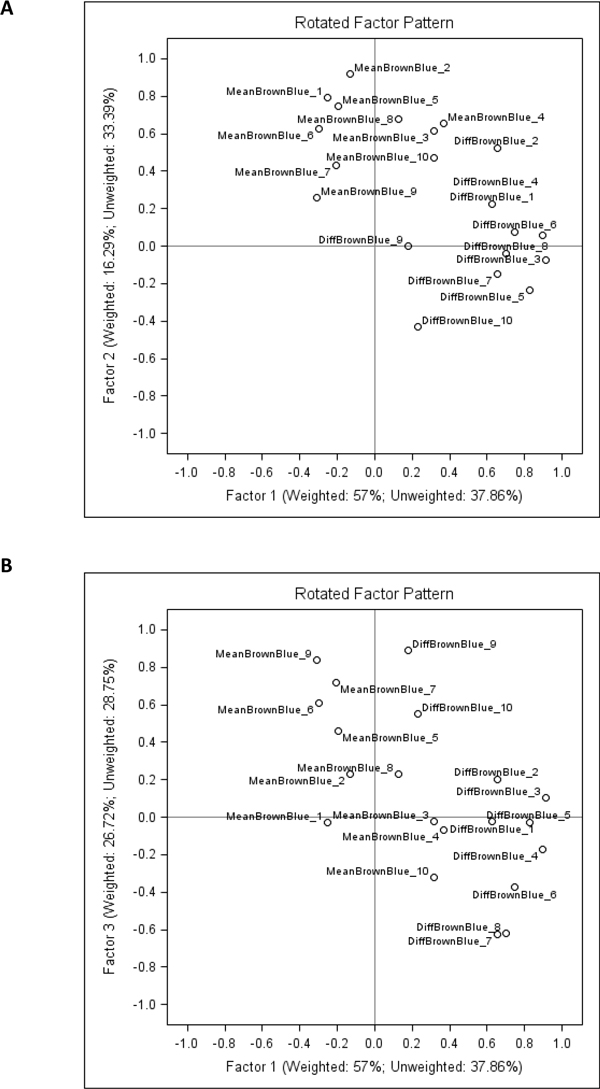
**Factor pattern representing parallel variance of the Colocalization and Genie/Nuclear algorithms in 10 individual TMA cores**. The variable loading plots of Factor-1 versus Factor-2: A. Factor analysis results from the Core#2; B. Factor analysis results from the Core#9.

**Figure 7:**
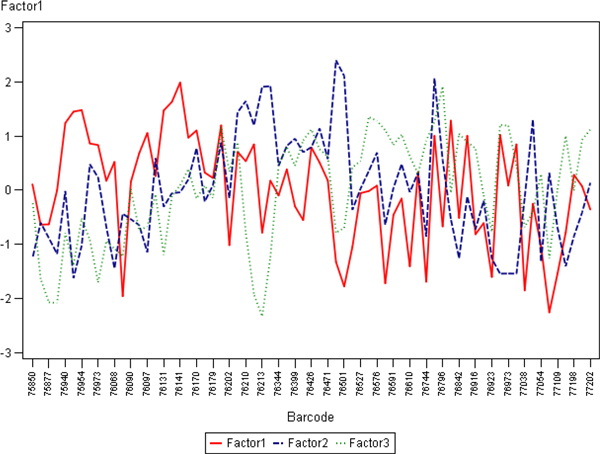
**Line plots representing slide-to-slide IHC staining variation of the Factor scores**. The factor scores generated from the analysis presented in Fig. 3 are plotted against the sequence of Ventana slide label ID labelled as Barcode) on the x axis.

Since the main feature to be extracted from the IHC tissue controls is Brown and Blue staining intensity (the variation is expected to be parallel to that of a test sample), we further concentrated on exploring the variation sources of the intensity variables in the individual cores, as presented in Figure 2F, G. The data were transformed to enable factor analysis on Brown and Blue intensity for each spot; furthermore, MeanBrownBlue Intensity ((Brown+Blue)/2) and DiffBrownBlue Intensity (Brown-Blue) were introduced to better contrast the absolute intensity and the colour balance variation. Indeed, factor analysis (Figure 6A) extracted Factor-1 characterized by positive loadings of DiffBrownBlue Intensity and Factor-2 characterized by positive loadings of MeanBrownBlue Intensity of the majority of the 10 cores. Since by definition these factors are independent, Factor-1 is expected to reflect Brown-Blue Intensity variation in opposite directions but parallel in the majority of the spots and represents the colour balance *per se*, mostly independent of the tissue-related variation. Factor-2 characterizes absolute intensity variation of both colours in the same direction, parallel in most spots, and therefore is likely to be dependent on tissue and/or scanning variations (section thickness, scanning regime, etc.). The pattern of the Factor-3 (Figure 6B) is somewhat peculiar: it is characterized by parallel variance of the MeanBrownBlue and DiffBrownBlue for the Core#9 and opposite variance of these variables for the Core#7. In other words, when Core#9 becomes darker it is because of deeper Brown, and vice versa, when Core#7 becomes darker it is because of deeper Blue. Importantly, the Factor-3 does reveal variable loading pattern for other cores, therefore, it is likely to express core-specific behaviour of the colour balance (with 2 extreme examples Core#7 and Core#9), thus can be interpreted as tissue-related variation which has been extracted as "noise" from the Factors 1 and 2. We therefore suggest that the Factor-1 scores provide a quantitative measure of Brown and Blue Intensity balance "purified" from the impact of tissue-related variation removed into the Factors 2 and 3. Consequently, slide-to-slide variation of the Factor scores can be monitored as depicted on Figure 7 and 7 further explored for quality assurance of digital IHC.

**Figure 8:**
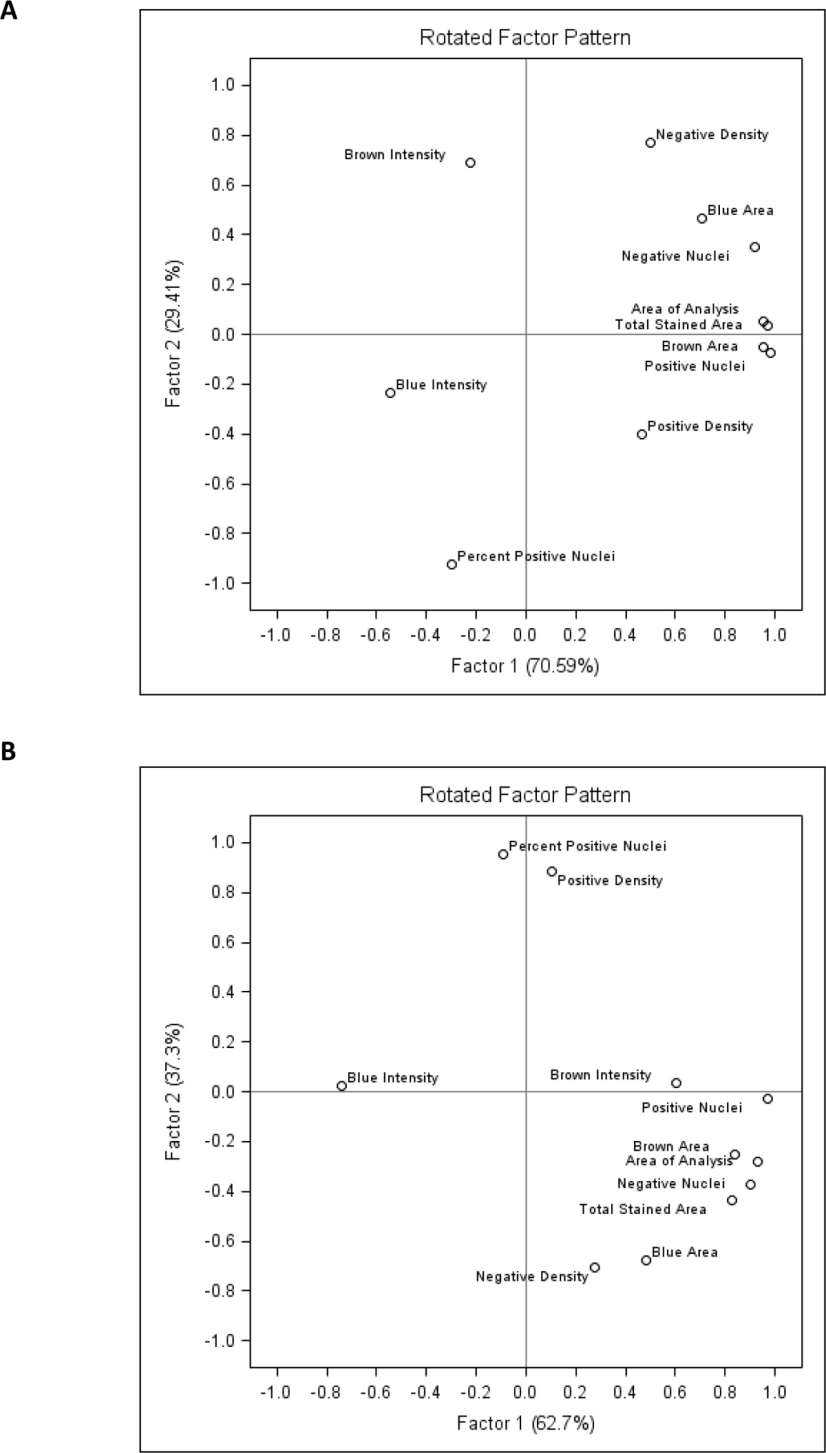
**Factor pattern representing parallel variance of the Colocalization and Genie/Nuclear algorithms in 10 individual TMA cores**. The variable loading plots of Factor-1 versus Factor-2: A. Factor analysis results from the Core#2; B. Factor analysis results from the Core#9.

Interdependencies between the Genie/Nuclear and Colocalization variables were further investigated by factor analyses performed for each individual tissue core. Although the factor patterns revealed some peculiarities for individual tissue cores, some common variance patterns could be generalized from the majority of the cores. As an example, a rather representative factor pattern of the Core#2 is plotted in the Figure 8A. Factor 1 was mainly represented by positive loadings of the variables expressing the epithelial cancer compartment size (analysis area, counts of positive and negative nuclei). Factor 2 reflected variation of the Percent of Positive Nuclei with opposite loadings of the Negative Density and Brown Intensity (less intense brown colour). Of note, more intense blue correlated with the Factor 1 in the Core#2, however, the loadings of Intensity variables were rather variable in different tissue cores. For comparison, similar factor pattern of the Core#9 is presented in the Figure 8B.

## Discussion

We aimed our experiments to explore the Ki67 IHC staining variation in our laboratory routine and to develop a prototype integration of the IHC tissue controls, tested by IA tools, and supported by highly automated statistical analysis processing to provide swift feedback on the results monitored. Although we did not pursue full automation with the monitor results available from our LIS at this stage, our approach enables this functionality and seamless integration into the routine workflow.

In general, we found that Ki67 IHC staining in our laboratory was rather stable overtime in terms of brown and blue staining intensity indicators, measured by Colocalization IA. Also, we could not demonstrate visually obvious variation of the staining intensity of the tissue controls overtime - unlike in our previous experiment with HER2 IHC where we found some notable aberrant colour variation, yet, escaping routine quality checks [7]. Nevertheless, our IA data revealed rather striking variation of Ki67-positive and negative nuclei counts and to some degree - the resulting percentage of Ki67-positive nuclei. Consecutive serial sections of 10 individual breast cancer tissue cores were used in our study to minimize possible impact of true tissue variation in the consecutive sections, which would be expected to be random in 10 unrelated samples.

Both individual and aggregated TMA core data from both Colocalization and Genie/Nuclear algorithms were analyzed to explore the sources of IHC staining variation. While Colocalization tool provides more direct and independent estimates of the staining intensity and colour balance, the Genie/Nuclear algorithm represents the actual IA result to be utilized further. Although we could not rule out tissue-related variation in our experiment, our findings raise a possibility of significant impact of blue color counterstain intensity on the performance of both IA algorithms, but most importantly - the Genie/Nuclear. It is likely that less intense blue counterstain decreases the blue area detected by the Colocalization tool, while the performance of the Genie and, potentially, the Nuclear algorithms is not sufficiently robust to detect proper amount of epithelial tissue mask and cell nuclei within the mask. Importantly, the range of blue intensity variation in our data was relatively low compared to that of the amount of tissue and cell numbers detected. Of note, our data reveal an association rather than a causal relationship between the variables; one needs to design more targeted experiments to obtain direct evidence.

The IA issues with hematoxylin counterstain, used routinely in IHC, have been highlighted, alternative counterstaining and IA techniques have been proposed [10-14]. Our experiment provides new data supporting the importance of further optimization and standardization of IHC procedures to achieve reliable processes and results with adoption of digital IHC. Interestingly, our data shed the light on how reproducible the Ki67 index would be in the consecutive sections of one 1 mm diameter core, if performed for research or clinical use. While variation of the percentage of Ki67-positive nuclei (the IA result) was satisfactory (standard deviation in all 10 cores ranged from 3 to 8, and relative error was within the range of 0.07 to 0.39, Table 1), the variation of cell numbers detected (the process) was higher. One may argue that the process variability needs to be dealt with, to achieve robust results by digital IHC techniques.

Inter-laboratory IHC staining variation is likely to be more significant and may impact visual estimation of Ki67 index: a recent international Ki67 reproducibility study [15] revealed unsatisfactory results of visual estimation which was even worse when the slides were stained locally. This implies significant inter-laboratory Ki67 IHC staining variability which should be considered when applying IA tools with unknown sensitivity to the staining characteristics. Although we have recently reported [9] on a methodology enabling accurate Ki67 index measurement in TMA samples by IA, the issue of IA calibration to possible inter-laboratory IHC staining variation and comparability of the Ki67 data between pathology labs remains open. One approach could be measuring signal-to-noise ratio of the images to evaluate quality before IA [16], however, adjustment of the images and/or analyses may require another effort. Ideally, IA tools should be robust and resistant to the IHC staining and scanning variations; however, this property requires further analysis and development efforts. As our study shows, one particular approach could be replacing the measurement of Ki67 index by the estimate of density of Ki67-positive cells in the tumour area: these two variables closely correlate; however, the Ki67-positive density does not rely on accurate detection of negative nuclei. This latter aspect may be especially relevant in the tumours with low Ki67 positivity.

## Conclusions

Our study presents a case for digital pathology solution to monitor staining of IHC multi-tissue controls by the means of digital IA, followed by automated statistical analysis procedures and integrated into the laboratory routine. We found that, even in consecutive serial tissue sections, tissue-related factors affected the IHC IA results; meanwhile, less intense blue counterstain was associated with less amount of tissue, detected by the IA tools.

## List of abbreviations

DiffBrownBlue: difference of Brown and Blue Intensity (Brown-Blue); IA: image analysis; IHC: immunohistochemistry; LIS: laboratory information system; MeanBrownBlue: mean of Brown and Blue Intensity (Brown+Blue)/2); TMA: tissue microarray; WSI: whole slide image.

## Competing interests

The authors declare that they have no competing interests.

## Authors' contributions

AiL, IB, JB, RM, and DLK designed and carried out the TMA IHC controls and digital image analysis workflow, edited the manuscript. AL, BP and PH performed statistical analyses and drafted essential parts of the manuscript. AL, DR, YI designed and constructed SQL-based integration of LIS, IA and statistical analysis systems. All authors participated in conception and design of the study, reviewing the analysis results, critically revised and approved the final manuscript.

## Authors' information

None.
